# Nutraceuticals and Functional Foods: A Comprehensive Review of Their Role in Bone Health

**DOI:** 10.3390/ijms25115873

**Published:** 2024-05-28

**Authors:** Maria Felicia Faienza, Silvia Giardinelli, Alessia Annicchiarico, Mariangela Chiarito, Barbara Barile, Filomena Corbo, Giacomina Brunetti

**Affiliations:** 1 Pediatric Unit, Department of Precision and Regenerative Medicine and Ionian Area, University of Bari “A. Moro”, 70124 Bari, Italy; mariafelicia.faienza@uniba.it (M.F.F.);; 2 Department of Medical Sciences, Pediatrics, University of Ferrara, 44121 Ferrara, Italy; 3 Department of Biosciences, Biotechnologies and Environment, University of Bari Aldo Moro, 70125 Bari, Italy; alessia.annicchiarico@uniba.it (A.A.); barbara.barile@uniba.it (B.B.); 4 Department of Pharmacy-Drug Sciences, University of Bari “A. Moro”, 70125 Bari, Italy; filomena.corbo@uniba.it

**Keywords:** nutraceuticals, functional foods, bone health, osteoporosis, polyphenols, carotenoids, polyunsaturated fatty acids, honey, tea, dried plums, blueberry, olive oil

## Abstract

Bone health is the result of a tightly regulated balance between bone modeling and bone remodeling, and alterations of these processes have been observed in several diseases both in adult and pediatric populations. The imbalance in bone remodeling can ultimately lead to osteoporosis, which is most often associated with aging, but contributing factors can already act during the developmental age, when over a third of bone mass is accumulated. The maintenance of an adequate bone mass is influenced by genetic and environmental factors, such as physical activity and diet, and particularly by an adequate intake of calcium and vitamin D. In addition, it has been claimed that the integration of specific nutraceuticals such as resveratrol, anthocyanins, isoflavones, lycopene, curcumin, lutein, and β-carotene and the intake of bioactive compounds from the diet such as honey, tea, dried plums, blueberry, and olive oil can be efficient strategies for bone loss prevention. Nutraceuticals and functional foods are largely used to provide medical or health benefits, but there is an urge to determine which products have adequate clinical evidence and a strong safety profile. The aim of this review is to explore the scientific and clinical evidence of the positive role of nutraceuticals and functional food in bone health, focusing both on molecular mechanisms and on real-world studies.

## 1. Introduction

Bone health is the result of the balanced activity between bone modeling and bone remodeling. The former is responsible for the longitudinal growth and mechanically induced adaption of bones, and it is mainly regulated by osteoblasts (OBs), whereas the latter replaces old and damaged bone with new bone and it is mainly regulated by osteoclasts (OCs).

These are lifelong processes regulated by genetic, hormonal, and environmental factors. Genetic factors impact skeletal development for approximately 60–80% of people [1] and numerous loci have been associated with low bone mass by Genome Wide Association (GWA) studies [2]. Environmental factors, such as diet, in particular, adequate intake of calcium and vitamin D, and physical activity, are responsible for the remaining 20–40% of people [1,3]. Changes in the hormonal status, and particularly the reduction in estradiol both in women [4] and men [5], are another great contributor to bone damage, with a great magnitude, especially in late adulthood.

OB and OC activity is a complex process regulated by cytokines, and it has been demonstrated that the receptor activator of nuclear factor kappa-light-chain-enhancer of activated B cells (RANK)/RANK ligand (RANKL)/osteoprotegerin (OPG) and WNT/β-catenin pathways plays a pivotal role in the control of osteoclastogenesis and osteoblastogenesis, respectively [6,7]. RANKL, through the binding to its specific receptor RANK, promotes differentiation and fusion of OC precursors and activates mature OCs to reabsorb the bone, by the activation of specific pathways, such as NF-κB, Nuclear Factor of Activated T Cells 1 (NFATc1), mitogen-activated protein kinases (MAPKs), and cFos. Mature cells express typical markers, including dendritic cell-specific transmembrane protein (DC STAMP), tartrate-resistant acid phosphatase (TRAP), cathepsin K, and matrix metalloproteinases (MMPs). OPG is a soluble decoy receptor secreted by OBs and bone marrow stromal cells which acts as an antagonist of RANKL, contrasting its osteoclastogenic effect [7].

On the other hand, the canonical Wnt/β-catenin pathway controls osteoblastogenesis and bone formation through several mechanisms including the renewal of stem cells, the stimulation of pre-OB replication, the induction of osteoblastogenesis, and the inhibition of OBs and osteocyte apoptosis [8,9]. This pathway promotes osteoblastogenesis through the activation of specific transcription factors, such as RUNX2 and osterix, which are required for the expression/synthesis of bone-specific alkaline phosphatase (BALP), collagen I, osteopontin, osteocalcin, and bone morphogenetic protein-2 (BMP2). Wnt signaling is modulated by various endogenous inhibitors including dickkopf-1 (DKK1) and sclerostin. DKK-1 blocks the maturation of OBs, decreases OPG levels, and increases RANKL expression, thus shifting the OPG/RANKL ratio in favor of bone resorption [10]. Sclerostin is a small protein produced by the *SOST* gene in the osteocytes that binds its receptors on the cell surface of OBs, activating a downstream intracellular cascade with the effect of inhibiting osteoblastic bone formation [11].

The activity of bone cells can be detected in the sera through specific markers. In detail, the typical systemic markers of OC activity are represented by C-terminal telopeptide of type I collagen (CTX), TRAP, and RANKL, whereas for OB activity, serum markers are osteocalcin, BALP, and procollagen type 1 N-propeptide (P1NP), etc.

Osteoporosis is the result of gradual deregulation of OB and OC activity, and although it is typically associated with aging, the contributing factors can already act during growth. Hence, the impairment of bone health can occur during the developmental age, when over a third of bone mass is accumulated, reaching a peak around the second decade of life.

Bone remodeling alterations have been observed in several congenital [12] and acquired pediatric disorders [13]. Particularly in children with obesity, the condition of chronic low-grade inflammation promotes OC activity by upregulating the production of RANKL and other inflammatory cytokines, and inhibiting osteoblastogenesis, thus disrupting the balance between bone formation and resorption in favor of the latter [14]. However, alteration of bone remodeling is most frequently assessed in adults with osteoporosis mainly associated with aging, but is also due to estrogen/androgen loss, glucocorticoid use, and bone metastasis.

Over the last few years, there has been a growing understanding and knowledge concerning human health and its association with foods. Additionally, the role of natural compounds, such as bioactive compounds with their possible function as health promoters, has been found to be very important thanks to studies performed using in vitro and in vivo animal models.

Nutritional supplements are commonly used in association with pharmacotherapy to prevent and treat osteoporosis [15]. Pharmacological treatment includes anti-resorptive drugs (e.g., bisphosphonates, estrogen replacement therapy, selective estrogen receptor modulators, calcitonin, denosumab, calcium and vitamin D supplementation), and anabolic drugs (e.g., teriparatide, abaloparatide, strontium ranelate, romosozumab) [16]. Nutrition plays a dominant role in skeletal health, both in achieving the highest BMD and in maintaining bone health [17]. Therefore, a balanced diet and good nutritional program can also prevent osteoporosis. The intake of macronutrients, vitamins, and minerals is often below the recommended values, especially if the disease is active [18]. Calcium (Ca) and vitamin D are the most frequently discussed nutrients with respect to BMD. In addition, nutraceuticals (e.g., resveratrol, anthocyanins, isoflavones, lycopene, curcumin, lutein, and β-carotene) and functional foods from the diet (e.g., honey, tea, dried plums, blueberry, and olive oil) can be efficient in supporting bone health [18].

Nutraceuticals are “foods or part of foods that provide medical or health benefits, including the prevention and/or treatment of a disease” [19].

Functional foods have numerous definitions: they can be defined as “processed foods having disease-preventing and/or health-promoting benefits in addition to their nutritive value” [20], but another accepted definition is that they are “foods that contain substances, in addition to nutrients, that may have potentially positive effects on health, beyond basic nutrition” *(FAO. FAO term portal. Available online at:* https://www.fao.org/faoterm/viewentry/en/?entryId=170967 *(accessed on 15 May 2022) (2022); Zeratsky K. Nutrition and healthy eating. Available online at:* https://www.mayoclinic.org/healthy-lifestyle/nutrition-and-healthy-eating/expert-answers/functional-foods/faq-20057816 *(accessed on 15 May 2022) (2022)).* The Academy of Nutrition and Dietetics includes among them both healthy natural foods and processed, fortified, enriched foods (*Ellis E. Functional Foods. Available online at:* https://www.eatright.org/food/nutrition/healthy-eating/functional-foods *(accessed on 15 May 2022) (2022).*

These terms often overlap with medical foods, probiotics, designer foods, pharmafoods, and dietary supplements, etc.

Nutraceuticals fall within the class of food supplements from a regulatory point of view and are currently not recognized as different from the latter. As their use has spread in recent years, leading to a huge increase in business around the world, there is urgency to redefine the concept and give precise rules. This will ensure that the available products have adequate clinical evidence and a strong safety profile with few unwanted side effects.

**Figure 1:**
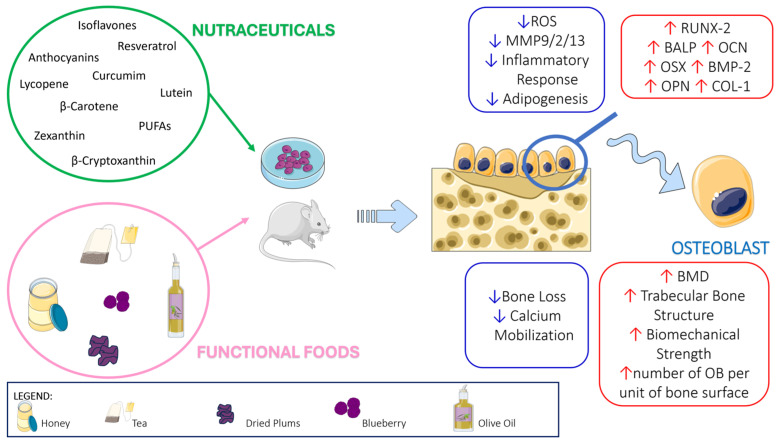
Functional foods and nutraceutical effects on OB formation and activity. In vitro and in vivo animal studies demonstrated that functional foods and nutraceuticals increase the levels of RUNX Family Transcription Factor 2 (RUNX-2), bone-specific alkaline phosphatase (BALP), Osteocalcin (OCN), Osteoblast-specific transcription factor Osterix (OSX), bone morphogenetic protein-2 (BMP2), Osteopontin (OPN), and collagen I (COL-1) and decrease ROS; Matrix Metalloproteinase-9/2/13 (MMP-9/2/13) intracellular levels. In addition, macroscopical effects on bone are evident as an increase in Bone Mineral Density (BMD), biomechanical strength, number of OB per unit of bone surface, and improvement of trabecular bone structure. The figure was generated using Servier Medical Art, provided by Servier, licensed under a Creative Commons Attribution 3.0 unported license.

**Figure 2:**
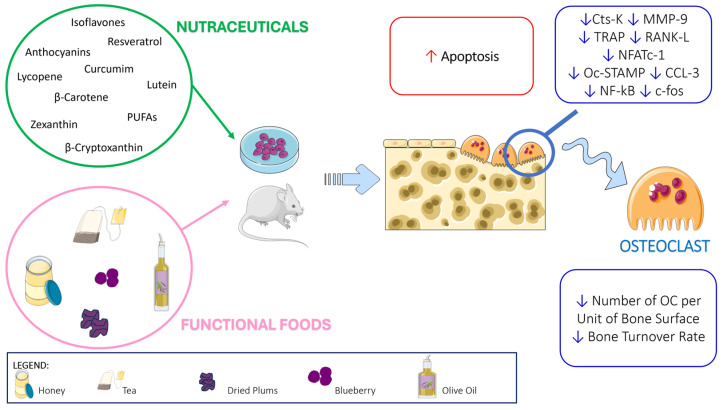
Functional foods and nutraceutical effects on bone formation and OC activity using in vitro and in vivo animal studies. These treatments reduce the molecular level of Cathepsin-K (Cts-K), Matrix Metalloproteinase-9 (MMP-9), Tartrate-Resistant Acid Phosphatase (TRAP), Receptor Activator of Nuclear Factor κ B Ligand (RANK-L), Nuclear Factor Of Activated T Cells 1 (NFATc-1), Osteoclast stimulatory transmembrane protein (Oc-STAMP), Chemokine C-C motif Ligand 3 (CCL3), Nuclear Factor kappa-light-chain-enhancer of activated B cells (NF-κB), and Protein c-fos. Functional foods and nutraceuticals reduce the number of OC per unit of bone surface and bone turnover rate. The figure was generated using Servier Medical Art, provided by Servier, licensed under a Creative Commons Attribution 3.0 unported license.

In light of this evidence, the aim of this review is to explore the scientific and clinical evidence of the positive role of nutraceuticals and functional food in bone health, focusing both on molecular mechanisms and on real-world studies, with the aim of providing a complete list of nutraceuticals and functional foods with adequate clinical evidence usable by everyone to improve bone health (Figure 1 and Figure 2 and Table 1, Table 2 and Table 3).

## 2. Nutraceuticals

### 2.1. Polyphenols

Polyphenols are a group of natural compounds largely found in various plant species. More than 8000 polyphenolic compounds have been characterized, and they can be classified into four main classes according to their basic chemical structure: phenolic acids, flavonoids, stilbenes, and lignans. Polyphenols show a multitude of positive effects on human health, mainly through immune modulation, but also through transcriptomic modulation. Indeed, it has been demonstrated that polyphenols are able to modulate the expression of genes involved in the development and progression of obesity [21]. It has been demonstrated that the bone impairment associated with obesity can be inhibited in vitro by polyphenols from sweet cherry extracts, through a reduction in TNF-α [22].

#### 2.1.1. Flavonoids

Flavonoids are the most abundant polyphenols in the human diet, and the most studied group of polyphenols; among their effects on human health, their anticancer, antioxidant, anti-inflammatory, antiviral properties, neuroprotective, and cardio-protective effects have been studied. Flavonoids can be divided into six subclasses: flavonols, flavones, flavanones, flavanols, anthocyanins, and isoflavones. Flavonoids are usually present in fruit and vegetables such as apples, citrus fruits, grapes, onions, berries, lettuce, and tomatoes [23]; see Table 1.

Other than anti-inflammatory properties, isoflavones (e.g., genistein and daidzein) also have estrogenic activity, and so they are commonly regarded to be phytoestrogens; due to this, their role in estrogen deficiency osteoporosis has been explored. Isoflavones are mostly found in legumes, and especially soybeans, chickpeas, fava beans, and nuts like pistachios and peanuts [24].

##### In Vitro and In Vivo Animal Studies

Isoflavones from chickpea sprouts administered in oral gavage (100 and 300 mg/kg/day) attenuate bone loss and improve trabecular microarchitecture and biomechanical properties of the fourth lumbar vertebra in ovariectomized (OVX)-induced osteoporotic rats, with a dose-dependent increasing trend. In addition, an enhancement of osteogenic differentiation of bone marrow stem cells (BMSCs), increased levels of OPG, and osteoclastic resorption inhibition via ERα modulation of the OPG/RANKL pathway have been demonstrated. RANKL-induced osteoclastogenesis was reduced under isoflavones from chickpea sprouts treatment [25].

##### In Vivo Human Studies

Isoflavones are effective in slowing down bone loss after menopause, as shown in a meta-analysis that included 18 studies for a total of 2350 postmenopausal women. The analysis showed that a daily intake of 106 (range, 40–300) mg of isoflavones for 6–24 months improved bone mineral density (BMD) in different sites, compared with controls. The effects of isoflavones on BMD might be associated with the treatment duration, ethnicity, time after menopause, form of supplements, and dose of isoflavones [26].

Recently, the effect of flavonoid subclasses intake on bone loss has been studied in 10,480 adults: the intake of flavones, isoflavones, and flavanones was positively associated with osteoporosis. In particular, a higher consumption of flavones and flavanones is significantly associated with a lower risk of bone loss at the femoral neck rather than the lumbar spine [27], although a specific cut-off of time and dose to achieve such beneficial effects remains to be clarified.

#### 2.1.2. Anthocyanins (Cyanidin)

Cyanidin is a pigment belonging to the flavonoid class of anthocyanins with a characteristic reddish-purple color; it can be found in many red berries, apples, plums, red cabbage, and red onion; see Table 1. Cyanidin has strong antioxidant and anti-inflammatory functions, as well as effects on bone health (Figure 1 and Figure 2).

##### In Vitro and In Vivo Animal Studies

Delphinidin, anthocyanidins present in berry fruits and vegetables, inhibited osteoclastogenesis and bone loss in osteoporosis murine models. Maqui berry extract (MBE, (0.25, 0.5, and 0.75 mg/day) positively affected bone remodeling both in vitro and in vivo. In detail, MBE promoted MC3T3-E1 differentiation towards OBs, increasing the mRNA levels of BMP2, Runx2, osterix, osteocalcin, and matrix extracellular phosphoglycoprotein. At the same time, MBE reduced OC formation and resorption. In vivo, MBE daily ingestion significantly enhanced trabecular bone and decreased the number of OCs per unit of bone surface, compared with vehicle controls in osteopenic mouse models [28]. Consistently, in vitro delphinidin positively affects BMSC differentiation towards osteogenesis and chondrogenesis, whereas it inhibits adipogenesis [29].

Other authors have reported that delphinidin treatment strongly inhibited OC formation from RAW264.7 cells with respect to the other anthocyanidins, cyanidin and peonidin. Delphinidin oral administration (10 mg/kg/day) prevented bone loss in both OVX and RANKL-induced osteoporosis model mice. Delphinidin inhibited the activity of key pro-osteoclastogenic transcriptional factors, such as NF-κB, c-Fos, and Nfatc1 [30].

Petunidin is another important anthocyanidin present in fruits, flowers, and seeds. It has been reported that Petunidin >5 μg/mL inhibited OC formation from RAW264.7 through the inhibition of c-Fos, NFATc1, MMP9, cathepsin K, and Dc-stamp. Conversely, petunidin (>16 μg/mL) promoted mineralization and increased the expression of osteocalcin and BMP2, whereas it inhibited MMP13, MMP2, and MMP9 in MC3T3-E1 cells. In sRANKL-induced osteogenic C57BL/6J mice, the daily ingestion of petunidin (7.5 mg/kg/day) enhanced bone quality, OB number, and OC number compared to untreated mice [31].

##### In Vivo Human Studies

Hardcaste et al. evaluated the effects of flavonoid intake in 3220 Scottish perimenopausal women; flavonoids consumption was estimated by the compilation of a food frequency questionnaire (FFQ) and 4-day food diary; among the 218 subjects who completed the study, the mean flavonoids consumption was 307 ± 199 mg/day. The authors found that procyanidin intake was positively associated with baseline and annual change in BMD at the femoral neck and lumbar spine [32]. Similar results were found by Welch et al., who demonstrated that a higher intake of anthocyanidins, estimated by FFQ, was associated with higher spine and hip BMD in 3160 women aged 18–79 years from the Twins UK adult twin registry [33]. Zhang et al. found a significant positive association between dietary intake of total flavonoids, flavan-3-ols, flavonols, flavones, proanthocyanidins and flavanones, and BMD in women, while the same result was not observed in men, probably due to the lower number of males recruited and to the estrogenic mimetic effects of flavonoids [34]. The effects of dietary anthocyanidins were also evaluated in a cross-sectional study involving 452 Chinese children aged 6–9 years; the authors found that anthocyanidin consumption, evaluated by an FFQ, was associated with higher bone mineral content (BMC) and BMD and these effects were more pronounced in boys than in girls [35].

#### 2.1.3. Stilbenes

Stilbenes are a class of polyphenols widely distributed in vegetal food (especially in grapes and peanuts); most of them act as antifungal phytoalexins in plants, compounds that are synthesized only in response to infection or injury.

Resveratrol is the most studied stilbene, and its presence has been reported in more than 70 plant species, such as in the skin of red grapes, mulberries, peanuts, and pines; see Table 1. It has well-known anti-inflammatory properties demonstrated both in vitro and in vivo, and independent effects of resveratrol on bone have been described.

##### In Vitro and In Vivo Animal Studies

Resveratrol improves bone quality in animal models of senile osteoporosis, estrogen deficiency-induced osteoporosis, and secondary osteoporosis by enhancing BMD, cortical and trabecular bone microstructure, bone strength, and bone histomorphometric parameters. The resveratrol administration route may differ from oral feeding and gavage to subcutaneous injection. It has been reported that resveratrol could affect osteoblastogenesis by activating the expression of SIRT1 [36].

Resveratrol has been tested in vitro for its effects on cell proliferation, viability, mineralization, and osteogenic genes expression in MC3T3-E1, human OB cell line, primary human, mouse, rat, and bovine OBs. In vitro studies demonstrated that resveratrol may directly induce OB differentiation and proliferation by increasing the levels of OB-specific genes, including Runx-2, osterix, type 1 collagen, BMP-2, osteopontin, and osteocalcin; see Figure 1.

In parallel, resveratrol enhances cell proliferation, collagen synthesis, ALP activity, and calcium deposition in OBs. Conversely, resveratrol reduces OC formation and activity. In detail, it has been reported that it reduces the expression of bone resorption markers in RAW264.7 cells such as cathepsin K and MMP9. Resveratrol (10^−5^ M) decreases the expression of the OC fusion marker Oc-stamp, RANK, TRAP, and cathepsin K by regulating the transcriptional activity of FOXO1, the PI3K/AKT [37] and NFATc1 signaling pathways [38]; see Figure 2.

##### In Vivo Human Studies

In a randomized placebo-controlled trial conducted on 74 middle-aged obese men with metabolic syndrome treated with 1.000 mg or 150 mg of resveratrol or with a placebo daily for 16 weeks, Ornstrup et al. found a dose-dependent increase in BALP and lumbar spine trabecular volumetric BMD, suggesting that resveratrol supplementation positively affects bone health by stimulating bone formation [39].

In a randomized, double-blind, placebo-controlled dietary intervention trial of 12 months duration, Corbi et al. evaluated the effects of dietary supplementation of 200 mg of fermented soy (including 80 mg of isoflavone aglycones and 10 mg of equol) and 25 mg of resveratrol from Vitis vinifera, in a group of 384 postmenopausal women compared with 38 women on a placebo treatment. The authors demonstrated an increase in bone formation markers such as osteocalcin and BALP, and a decrease in bone resorption markers such as deossypiridinoline and TRACP-5b, associated with the improvement of BMD in the supplemented group when compared with the placebo one [40]. In agreement with these results, the Resveratrol for Healthy Aging in Women (RESHAW) trial demonstrated that supplementation with a low dose of resveratrol (75 mg twice daily) in postmenopausal women during a 1-year study improved the BMD in the lumbar spine and neck of the femur when compared with the placebo group. Moreover, these effects were also associated with lower serum CTX levels [41]. All studies are reported in Table 3.

#### 2.1.4. Curcumin

Curcumin is a lipophilic polyphenol contained in turmeric, a common spice that comes from the root of *Curcuma longa*; see Table 1. It is commonly used as a coloring and flavoring agent in many cuisines. The effects of curcumin on the musculoskeletal system, as well as on osteoporosis, were first established by both in vivo and in vitro studies and recently in humans.

##### In Vitro and In Vivo Animal Studies

Different studies have shown the protective role of curcumin in bone health [42]. It can sustain osteoblastogenesis and inhibit osteoclastogenesis, thus preserving bone health [43].

In detail, curcumin stimulates the proliferation of OBs and increases the levels of bone formation markers such as BALP, osteocalcin, and Runx2 [44,45]. It also stimulates β-catenin translocation in the nuclei with osteoprotective effects [46]. As an antioxidant, curcumin may scavenge free radicals and ROS activity with osteoprotective effects [47]. After curcumin treatment, the ROS levels decreased and osteoblastogenesis increased in MC3T3 following long-term exposure to microgravity [48]. Dai et al. reported that curcumin reduces oxidative stress-induced apoptosis in OBs through Akt to inhibit the phosphorylation of Gsk3β, a serine/threonine kinase [49]. In turn, Gsk3β reduced the levels of nuclear factor-like 2 (Nrf2), a crucial transcription factor for cellular antioxidant security implicated in apoptosis [50]. Consistently, Li et al. showed that curcumin rescued MC3T3-E1 from oxidative stress-mediated damage through the inhibition of the Gsk3β/Nrf2 signaling pathway [51]; see Figure 1.

Additionally, curcumin affected osteoclastogenesis through different mechanisms. In detail, curcumin inhibited the differentiation of pre-osteoclast into mature OCs by decreasing CCL3 levels [52]. Li et al. reported that curcumin inhibited osteoclastogenesis markers such as MMP-9, MMP-13, and cathepsin K by an increase in miR-365 [44]. In a co-culture system, curcumin decreased osteoblastogenesis by inhibiting IL-1α-induced RANKL expression in osteoblastic precursors [53]. Park et al. demonstrated the anti-osteoclastogenic effect of curcumin on RAW264.7 cells [54]; see Figure 2.

Interestingly, Ke et al. reported that in rats, the molecule (110 mg/kg, oral administration for 60 days) prevented bone loss by blocking RANKL stimulatory action on OC autophagy [55]. The curcumin osteoprotective action is mediated by the inhibition of bone resorption pathways, such as the NF-κB and MAPK pathways [56,57].

Interestingly, results arose from curcumin administration in different animal models of bone loss, such as ovariectomy (OVX), glucocorticoid-induced osteoporosis (GIO), diabetic osteoporosis, and microgravity-induced bone loss. In detail, using a murine OVX model, Kim et al. showed that continuous treatment for 8 weeks with curcumin (9.5 μg/g/d given intragastrically through an esophageal cannula) significantly rescued OVX-induced bone loss through an increase in GPX-1 antioxidant activity and a decrease in OC activity [56]. Liang et al. reported that in OVX mice oral curcumin (200 mg/kg/d) reversed OVX-trabecular bone loss [52]. Using a mature rat model of OVX-mediated osteoporosis, different studies have demonstrated the positive effect of high doses of curcumin (administered orally or by a stomach tube) on bone strength and turnover [58,59].

Using a rat model of GIO, Chen et al. demonstrated that curcumin treatment enhanced femoral BMD, and ameliorated trabecular bone structure, and OB viability. The same authors reported that curcumin’s positive effect in GIO is linked to miRNAs [60], inhibition of the OPG/RANKL/RANK pathway [44], and modulation of the Wnt/β-catenin pathway [61], injected i.p, i.m, and subcutaneously, respectively. Curcumin displayed an osteoprotective role by restoring OBs damaged by high glucose and supporting BMSC osteogenic differentiation [62,63].

Fan et al. reported that in a high-glucose environment pretreatment with curcumin promoted BMSC-mediated osteogenic differentiation and angiogenic coupling, thus preventing diabetes-induced bone loss through inhibition of the NF-κB pathway. In addition, in vivo studies showed that curcumin prevented bone loss in diabetic osteoporotic mice [64]. In detail, in diabetic rats fed on a standard diet with 0.5% curcumin, the molecule inhibited osteoclastogenesis [65]. In addition, Liang et al. found that in type 2 diabetic osteoporotic rats oral curcumin treatment protected the microarchitecture of bone and enhanced bone mechanical properties by modulating the TGFβ/Smad2/3 pathway [66]. Furthermore, in rats, curcumin (via daily oral gavages) positively affected microgravity-induced bone loss by preventing reactive oxygen species synthesis as well as osteoclastic marker expression [48].

##### In Vivo Human Studies

Khanizadeh et al. evaluated the effects of curcumin supplementation in a randomized, double-blind trial study involving 60 osteoporotic postmenopausal women of whom 20 were receiving alendronate, 20 alendronate + 110 mg/day of curcumin, and 20 were controls. In the alendronate + curcumin group, at the end of the study, the authors found that BALP and CTX serum levels significantly decreased, and BMD indexes increased significantly in four areas at the end of study compared to the other groups [67]; see Table 3.

In another study, curcumin supplementation at a dose of 110 mg/kg for 6 months was associated with lower BALP levels and higher mean BMD values of the femoral neck and hip in patients with spinal cord injuries [68]; see Table 3.

### 2.2. Carotenoids

Carotenoids are organic pigments largely widespread in nature, particularly in plants, fruits, vegetables, fungi, bacteria, algae, and fish. They have robust antioxidant properties and act as scavengers of radical oxygen species and alternative free radicals of various origins. Current knowledge on carotenoids indicates that they can also have protective effects on bone health; lycopene, β-carotene, lutein, zeaxanthina, and β-cryptoxanthin are the carotenoids with most evidence in humans.

#### 2.2.1. Lycopene

Lycopene is a bright red carotenoid hydrocarbon found in red fruits and vegetables, mainly in tomatoes but also in red carrots, watermelons, grapefruits, and papayas; see Table 1. Lycopene, together with β-carotene, is one of the most commonly used carotenoids in scientific research in relation to the beneficial effects on bone health.

##### In Vitro and In Vivo Animal Studies

Rao et al. in vitro demonstrated that the water-dispersible, lycopene microemulsion preparation inhibited OC formation and activity [69]; see Figure 2. Russo et al. proved that 5- and 10-µM lycopene inhibited RANKL expression on osteoblastic Saos-2 cells, whereas it simultaneously increased collagen I and RUNX2 expression, without affecting OB proliferation [70]. Ardawi et al. supported these results by reporting that lycopene nanoparticles increased OB differentiation with enhanced ALP and osteocalcin secretion. Other authors showed that ≥500 nM lycopene stimulated OB proliferation and differentiation, with simultaneously reduced osteoclastogenesis [71]; see Figure 1.

Additionally, Bengi et al. showed that in the human OB cell line (CRL-11372), lycopene exerts a proliferative effect [72]. In animal models, 10- or 20-mg/kg lycopene, which was administered once orally using an intragastric feeding tube, inhibited BMD decrease in OVX rats, and increased lumbar and femoral BMD, even if these differences did not reach the statistical significance. Furthermore, treated rats displayed higher Ca and phosphorus serum levels, compared with controls [73]. The lycopene-based dietary intervention ameliorated the biomechanics and trabecular quality of bone, thus suppressing the increase in bone turnover induced by OVX [73].

Interestingly, Iimura et al. reported that in rats, lycopene consumption after the OVX decreased sera and urine bone resorption markers. Furthermore, tibial BMD was augmented [74]. The authors reported that although oxidative stress parameters did not significantly change, they correlated with tibial BMD. Oliveira et al. have reported that administering by gavage a 10-mg/kg lycopene to OVX rats for 60 days delays femoral bone loss, through beneficial effects on OBs [75]. Consistently, Ardawi et al. reported that a 12-week lycopene supplementation diet caused the simultaneous promotion of OB function and inhibition of osteoclastogenesis [76]. In detail, the authors reported that in bone marrow cell lysates, all lycopene doses reduced RANKL, while higher lycopene doses reduced OPG. OB differentiation markers were significantly augmented; moreover, lycopene treatment increased the relative expression of all antioxidative stress enzymes through the regulation of AGE/RAGE/NF-κB signaling. Lycopene consumption is associated with the improvement of cortical and trabecular bone as well as biomechanical properties. The effect is maximized using a 45 mg/kg body weight/day supplementation [76]. These data were supported by Xia et al. showing that in obese mice lycopene improves femoral quality and prevents bone microarchitecture weakening [77]. Liang et al. also showed the beneficial effect of diet with lycopene supplementation on bone quality and strength in OVX-rats through osteoclastogenesis inhibition, possibly through the downregulation of IL-6 levels [78]. Moreover, Iimura et al. reported that a rat diet with 100-mg/kg lycopene caused an increase in lumbar spine and tibial metaphysis BMD with an improvement of bone turnover markers, without affecting oxidative stress pathway [79]. Consistently, Semeghini et al. reported that daily ingestion 10 mg/kg lycopene for 8 weeks after OVX enhanced the OB and osteocytes total number in femora. Simultaneously, rat fed lycopene displayed a decrease in OC volume and number [80].

##### In Vivo Human Studies

Data from the Framingham Osteoporosis Study, involving 370 men and 576 women, show that high lycopene intake is associated with a lower risk of hip and non-vertebral fractures. A beneficial effect on reducing the risk of hip fracture was found with a mean intake of 12.66 mg per day of lycopene [81].

Rao et al. evaluated the effects of lycopene consumption on bone health in 33 postmenopausal women; lycopene intake was estimated by serum dosage and by FFQ and the authors found that higher lycopene serum levels were associated with lower serum levels of cross-linked amino-terminal N-telopeptide (NTx), a marker of bone resorption, and less protein oxidation [82].

#### 2.2.2. β-Carotene

β-Carotene is a strongly colored red-orange pigment abundant in carrots, pumpkin, spinach, and sweet potatoes (Table 1), with antioxidant and anti-inflammatory properties. It is a provitamin A compound, converting in the body to retinol (vitamin A).

##### In Vitro and In Vivo Animal Studies

Wang et al. demonstrated that β-carotene inhibited OC formation and activity, through the suppressed increase in c-Fos, NFATc1, and cathepsin K [83]. Furthermore, they reported that β-carotene reduced the viability of bone marrow-derived monocytes and macrophages; see Figure 2. In turn, Nishide et al. showed that β-carotene affected osteoblastogenesis, upregulating BALP activity in MC3T3-E1, and β-carotene treatment enhanced Runx2, osteopontin, and BALP expression [84]; see Figure 1. Matsumoto et al. demonstrated that a 3-week β-carotene supplementation (0.025%) reduced bone impairment in hind limb suspended mice, an unloading model [85]. Consistently, they found that in the bone marrow cells, OPG levels increased in mice fed with a β-carotene-supplemented diet [85].

##### In Vivo Human Studies

Handel et al. reported that the serum β-carotene concentrations of pregnant women may impact bone mineralization in the offspring. Indeed, they found that increased maternal serum levels of β-carotene in late pregnancy were positively related with the offspring total-body BMC and the total-body bone area [86]. Kim et al., in a cross-sectional study conducted in postmenopausal Korean women, found that β-carotene intake, estimated by the compilation of a semi-quantitative FFQ, was significantly associated with a higher lumbar spine, femoral neck, and total hip T-scores, suggesting that β-carotene may constitute a crucial dietary component positively associated with BMD [87]. Ambrosini et al. analyzed 2322 adults, finding that the cumulative supplementation of β-carotene (30 mg/day or 0.75 mg/day over a period of 1 to 16 years) resulted in a marginal reduction in the risk osteoporotic fracture in men [88]; see Table 3.

In a recent metanalysis, the intake of β-carotene was negatively associated with the risk of osteoporosis in both male and female subgroups. This association was also found in the Asian subgroup whereas no association was observed in the Western subgroup, suggesting that the beneficial effects of β-carotene on bone health could be influenced by ethnic variability even if the underlying mechanisms are not known [89].

#### 2.2.3. Lutein and Zeaxanthin

Lutein and zeaxanthin are the most common xanthophylls in green leafy vegetables (e.g., kale, spinach, broccoli, peas, and lettuce) and egg yolks; see Table 1.

##### In Vitro and In Vivo Animal Studies

Tominari et al. demonstrated that in murine bone marrow cell cultures, lutein decreased OC activity, through the inhibition of the interleukin-1–induced osteoclastogenesis and OC survival [90]. Furthermore, they reported that in OB cultures, 30 µM lutein treatment stimulated BMP2 expression and decreased sclerostin mRNA levels; see Figure 1. Takeda et al. showed that a 4-week oral administration of 1% lutein caused a significant BMD and bone section area improvement in femoral cortical bones of 5-week-old male mice [91]. Li et al. reported that 50 mg/kg lutein reduced oxidative stress and inflammation in sera and femurs of OVX rats compared with the sham-operated. A lutein anti-inflammatory effect was demonstrated by the decrease in IL-6, IL-8, and TNF-α serum levels [92]. Moreover, a significant increase in the OC-specific marker NFATc1 was found in OVX rats compared to sham rats [92]; see Figure 2.

##### In Vivo Human Studies

Zeaxanthin exhibits antioxidant properties that could exert protective effects on bone health. Niu et al. found a positive correlation between high blood zeaxanthin levels and femoral neck strength. Moreover, a positive correlation was found between the blood zeaxanthin level and other bone strength parameters, such as bending strength index, compression strength index, and impact strength index [93]. In another study, Murphy et al., analyzing 4513 community-dwelling adults aged >50 years, found that subjects with higher lutein and zeaxanthin serum levels were less likely to develop frailty after 8 years of follow-up. Baseline plasma lutein and zeaxanthin concentrations were also positively associated with several indices of musculoskeletal health [94].

#### 2.2.4. β-Cryptoxanthin

β-cryptoxanthin is a β-carotene derivative and in the human body it becomes retinol and, similarly to β-carotene, it exerts antioxidant, anti-obesity, anti-inflammatory, and anticancer activities as well as the prevention of bone tissue loss [95]. The main sources of β-cryptoxanthin are pumpkins, persimmons, chili peppers, tangerines, and papaya [96]; see Table 1.

##### In Vitro and In Vivo Animal Studies

Positive β-cryptoxanthin effects on bone metabolism were reported in different studies using bone cell lines, preosteoclasts, pre-osteoblasts, and periodontal ligament cells as well as bone tissues derived from femoral diaphyseal, metaphyseal tissues, and calvariae; see Figure 1 and Figure 2. Addition of 10^–7^ and 10^–6^ M β-cryptoxanthin to MC3T3-E1 caused a proliferation together with an enhancement of the concentration of proteins, DNA, BALP, and Ca, indicating a significant β-cryptoxanthin effect on osteoblastogenesis. Treatment of MC3T3-E1 with 10^–6^ M β-cryptoxanthin enhanced the levels of Runx2, collagen-I, and BALP. Different authors demonstrated that β-cryptoxanthin had a stimulatory action on OB transcriptional activity [97,98,99]. Additionally, β-cryptoxanthin augmented OPG expression, thus preventing bone resorption in periodontitis models [100]. β-Cryptoxanthin (10 µM) decreased NF-κB activity in MC3T3-E1 pre-osteoblasts [100]. NF-κB antagonized the suppressor of mothers against decapentaplegic (SMAD) activation, caused by BMP2 and transforming growth factor β1 (TGF-β1). β-cryptoxanthin inhibited NF-κB activity, thus increasing the activation of SMAD, a crucial pathway of lineage commitment towards osteoblastic differentiation. β-Cryptoxanthin increased TGF-β1-induced SMAD activation [101]. Furthermore, β-Cryptoxanthin inhibited bone resorption in vitro [102]. β-Cryptoxanthin induced apoptosis and inhibited OC formation from mouse marrow culture. OC apoptosis occurred both with or without MCSF and RANKL through a β-Cryptoxanthin-mediated increase in proapoptotic proteins. The same OC cultures displayed a decreased expression of TRAP and cathepsin K [103]. LPS (1 ng/mL) induced osteoclastogenesis in the co-cultures of OBs and murine bone marrow cells. A dose of 1 to 5 µM of β-cryptoxanthin in a dose-dependent manner decreased osteoclastogenesis in LPS-treated co-cultures of murine bone marrow cells and OBs [104]. It has been reported that β-cryptoxanthin inhibited osteoclastogenesis through the suppression of the NF-κB pathway [105]. β-Cryptoxanthin affected bone formation in healthy animals and in animal models of bone loss, such as osteopenia, osteoporosis, periodontitis, and diabetes. In detail, Yamaguchi et al. described the β-cryptoxanthin (oral administration) anabolic effects on rat bone [106], which were associated with a higher mineralization degree, an increased DNA concentration (index of bone cell number), and ALP activity. Using an OVX-induced estrogen deficiency in rats, it was found that β-Cryptoxanthin administered to rats daily for 3 months improved the polar strength–strain index and avoided morphological changes in the femora [107]. Ozaki et al. reported that β-Cryptoxanthin, dissolved in water and orally administered to OVX mice at concentrations of 1 mg/L and 10 mg/L for 28 days, reduced OC activity and bone volume, without affecting bone formation, suggesting that effect of β-cryptoxanthin on bone volume prevented OC activity, rather than osteoblastic function [105]. Furthermore, in a mouse model of periodontitis, β-cryptoxanthin injection inhibited rescued alveolar bone loss induced by LPS [104]. Uchiyama et al. evaluated the activity of β-cryptoxanthin (0.005 or 0.01 mg/100 g for 14 d) on diabetic rats, showing that oral administration of β-Cryptoxanthin improved bone health in treated rats by enhancing bone formation and inhibiting OC activity [108].

##### In Vivo Human Studies

A group of 21 healthy participants (10 men and 11 women) received 192 mL of juice from Satsuma mandarin, containing 1540 mg of β-cryptoxanthin, or reinforced juice, containing 2880 mg of β -cryptoxanthin. The juice consumption was found to increase the concentration of γ-carboxylated osteocalcin, which stimulated bone formation; the reinforced juice was also found to decrease the levels of TRAP and N-telopeptide of type I collagen [109]. Yamaguchi et al. evaluated the effects of β-cryptoxanthin-supplemented satsuma mandarin juice, at concentrations of 1.5, 3.0, and 6.0 mg/200 mL, on healthy adult volunteers including 19 men and 71 women (35 premenopausal and 36 postmenopausal). Serum levels of β-cryptoxanthin in subjects consuming juice were significantly augmented and maintained for 28 days after the interruption of juice consumption. The juice augmented the values of bone formation markers (BALP and γ-carboxylated osteocalcin) and decreased the values of markers indicating bone resorption (TRAP and N-telopeptide of type I collagen) both in men and women [110]. These data have been supported by a recent meta-analysis of observational studies demonstrating that high dietary intake of β-cryptoxanthin correlated with a lower risk of osteoporosis and occurrence of hip fractures [111].

### 2.3. Polyunsaturated Fatty Acids (PUFAs)

PUFA lipids are polyunsaturated fatty acids widely distributed in nature, that include the well-known Omega 3 (ω-3) and Omega 6 (ω-6). They are referred to as “essential fats” since the human body cannot produce ω-3, and primary dietary sources are fish, algae, plant oils (walnuts, edible seeds, and flaxseeds as well as hempseed oil); see Table 1.

#### 2.3.1. In Vitro and In Vivo Animal Studies

In a mouse model of GIO, it has been demonstrated that oil from Antarctic Krill that is rich in ω-3 eicosapentaenoic acid (EPA) and docosahexaenoic acid (DHA) augmented Runx2 expression, thus promoting osteoblastogenesis, whereas it decreased PPARγ levels and thus adipogenesis [112]. Additionally, Cugno et al. reported that EPA and DHA-rich fish oil offered safeguarding effects against the reduced osteoblastogenesis and the heightened differentiation of adipocytes in MSCs induced by rosiglitazone. Rosiglitazone is an antidiabetic agent known in bone marrow to elevate bone resorption and adiposity [113].

Consistent with the in vitro results, an animal study agreed that fish oil downregulated the expression of PPARγ in rosiglitazone-treated mice. Collectively, these findings suggested that the fate of MSCs toward adipogenesis or osteogenesis is influenced by the source of fatty acids. Specifically, ω-6 arachidonic acid (AA) and prostaglandin E2 (PGE2) stimulate adipogenesis by activating a PPARγ-dependent pathway [114], and decreasing Runx2, whereas DHA and EPA induce osteogenesis through the same pathways.

Kasonga et al. demonstrated that in vitro EPA and DHA activated both PPARγ and PPARα in CD14+ monocytes, thus inducing osteoclastogenesis [115]. Polyunsaturated fatty acids (PUFAs), acting as PPAR ligands, could influence osteoclastogenesis via PPARs.

Nakanishi et al. demonstrated that DHA and EPA treatment in MSCs indirectly inhibited osteoclastogenesis by suppressing the transcriptional activity of NF-κB via PPARγ binding. This NF-κB transcriptional activity inhibition led to a decrease in the expression of NF-κB-regulated genes such as IL-6, TNFα, and COX2, resulting in a reduction in RANKL levels [116]. Nonetheless, fatty acids stimulate osteoclastogenesis through the increased expression of the c-Fos gene, which is PPARγ-mediated [116].

Watkins et al., using male rats, demonstrated that a diet rich in PUFA can influence bone formation, as increasing the ratio of ω-6/ω-3 PUFA in the diet leads to an increased the arachidonic acid/EPA ratio in bone organ culture, and this in turn determines the capacity to synthesize PGE2 [117].

Evidence indicates an interplay between ω-3 PUFA and estrogen through the observation of mitigated bone mineral loss in ovariectomized rats administered with EPA [118]. This finding suggests a potential bone-preserving effect of EPA, possibly due to a reduction in bone resorption or enhancement of bone formation.

In 2023, Wang et al. studied the role of EPA in vitro on OB and OC differentiation of mouse BMSCs and BMMs, respectively. High-dose EPA promotes osteoblastogenesis in an inflammatory environment, as well as upregulates the expression of OB-specific proteins and genes. EPA is able to restore the reduced osteogenic capacity of mBMSCs caused by TNFα-induced inflammation, and to rescue the OB/OC balance via regulation of RANKL and OPG expression in OBs. The in vivo effects of EPA were determined using an OVX mouse model treated with EPA or a vehicle. EPA prevented bone loss in OVX mice, suggesting the potential application of EPA in postmenopausal osteoporosis [119].

Farahnak et al. examined the dose–response relationship of DHA on bone characteristics in 6-week-old female rats for 10 weeks [120]. DHA was incorporated into a standard chow diet at concentrations ranging from 0.1% to 1.2% *w*/*w*. The findings demonstrated a strong increase in whole-body BMD across all groups administered with DHA compared to the control group. Furthermore, a significant increase in the peak force required for bone fracture was evident. Consistently, Chen et al., using *fat-1* transgenic mice able to endogenously convert ω-6 to ω-3 PUFAs, a particular model allowing us to distinguish ω-3 PUFA’s role, showed a significant acceleration in fracture healing, suggesting that ω-3 PUFAs enhance endochondral ossification and accelerate the remodeling of calcified calluses after fracture [121].

#### 2.3.2. In Vivo Human Studies

Several studies in humans demonstrate that regular consumption of long chain ω-3, polyunsaturated fatty acids (LCO3-PUFA) results in health benefits. Few studies have investigated the effects on bone, with conflicting results [122,123]. Lavado-Garcìa et al. examined the effects of LCO3-PUFA and bone metabolism in a cohort of 1865 Spanish women aged 20–79 years, finding that higher consumption of LCO3-PUFA was positively associated with BMD at the hip in normal and osteopenic women and in normal women with lumbar BMD, even if no such association was found in osteoporotic subjects [122]. Feehan et al. evaluated 300 postmenopausal women, finding that a low ω-6: ω-3, ratio was associated with higher femur BMD and a higher ω-3, LCPUFA was associated with lower markers of bone resorption [123].

**Table 1:** Main sources of nutraceuticals and effective dosage on human bone health.

Nutraceutical	Main Source	Effective Dosage	Reference
Isoflavones	Legumes (including soybeans, chickpeas, fava beans), nuts (pistachios, peanuts)	106 mg/day	[26]
Cyanidin	Red berries, apples, plums, red cabbage, and red onion	Not available	[32,33,34,35]
Resveratrol	Skin of red grapes, mulberries, peanuts, and pines	75 mg twice/day	[41]
Curcumin	Turmeric (curcuma longa)	110 mg/day	[67,68]
Lycopene	Tomatoes, red carrots, watermelons, grapefruits, and papayas	12.66 mg/day	[81]
β-carotene	Pumpkin, spinach, sweet potatoes	30 mg/day	[88]
Lutein and zexanthin	Green leafy vegetables (kale, spinach, broccoli, peas, and lettuce), egg yolks	Not available	[93,94]
β-cryptoxanthin	Pumpkins, persimmons, chili peppers, tangerines, papaya	2880 μg/day	[109]
PUFAs	Fish, algae, plant oils (walnuts, edible seeds, flaxseeds, hempseed oil)	Not available	[122,123]

## 3. Functional Foods with Impact on Health

### 3.1. Honey

Honey is a sweet and viscous substance, made by and stored to nourish bee colonies, widely used in cooking, baking, and as a sweetener. Honey is particularly rich in natural macro- and micronutrients [124] and a wide range of minor constituents with beneficial properties. Among them, flavonoids and phenolic acids, which can be found in honey (Table 2), act on several signalling pathways, resulting in antiresorptive and anabolic effects [125]. From the group of polyphenols, kaempferol, quercetin, and luteolin have an anti-osteoporotic function [126]. In addition, vitamin D3 and its hydroxyderivatives can be detected in honey, contributing to its antioxidant properties [127,128], and it has also been hypothesised that honey can have a role in boosting calcium absorption, as shown in rat models [129].

#### 3.1.1. In Vitro and In Vivo Animal Studies

In heat-stressed broiler chickens, tibial BMD and calcium content were increased by the addition of 20 mL/L honey to drinking water starting from day 28 until day 56 [15], suggesting that honey ameliorates broiler calcium metabolism [130]. Other authors evaluated the long-term consequences in rats fed the honeydew honey (10% *w*/*w*) diet for one year compared to a sugar-free and sucrose diet [131]. BMD was enhanced in the honey-fed rat group with respect to the sugar-free diet group [131]. BMD was unchanged comparing the honey- and sucrose-fed rats.

Ramli et al. showed that in rats, GIO is alleviated following treatment with Kelulut honey (a type of Malaysian honey that is principally made by stingless bumblebees) for 2 months at doses of 200 mg/kg/day and 400 mg/kg/day [132]. This effect is due to its antioxidant activity. Consistently, honey treatment improved trabecular bone quality together with OB number with a simultaneous reduction in OCs. Ekeuku et al. [133] investigated the outcomes of Kelulut honey supplementation (1 g/kg for 8 weeks) on the bone health of rats with metabolic syndrome, known to be linked to bone loss [134]. Treated rats showed a significant decrease in OC on the bone surface with respect to the control group [133]. Consistently, Mohd Ramli et al. [135] reported that honey can be used in the treatment of metabolic syndrome and associated comorbidities.

Abu-Serie et al. [136] reported the positive effect of a jointed extract of honey and Greek thyme (*Thymus vulgaris*) on hydrocortisone-induced osteoporosis, ameliorating bone turnover, inflammation, and oxidative stress in vitro. Interestingly, a stronger anti-osteoporotic effect was reported with respect to alendronate, a common bisphosphonate.

Tualang honey is made by *Apis dorsett* bees, as in tropical rainforests they construct beehives on the Tualang tree. Two-week administration of Tualang honey at doses 0.2 g/kg and 1.0 g/kg by oral gavage to OVX rats significantly restored the trabecular thickness of the tibial bones compared to the untreated rats. Interestingly, the low dose (0.2 g/kg) of Tualang honey augmented serum-free testosterone concentration in OVX rats, thus exerting a protective effect against bone loss. It is uncertain whether Tualang honey supported the transformation of estrogen into androgen [137]. In an additional study, rats receiving Tualang honey dissolved in water at 0.2 g/kg for six weeks displayed better bone structural parameters, particularly for the trabecular bone [138].

Manuka honey in combination with α-cyclodextrin (Manuka Honey with Cyclopower™ (MHCP)) was found to increase honey delivery and to improve its water stability and solubility [139,140]. Katsumata et al. reported that in OVX mice MHCP in the diet decreased the CTX serum levels, femoral RANKL, and NFATc1 expression. These findings suggested that the MHCP had prebiotic effects which increase the action of honey by reducing bone resorption in OVX mice by suppressing inflammation [141].

Tualang honey supplementation at dosages of 2 g/kg and 4 g/kg for 12 weeks enhanced the biomechanical strength of the right femurs in OVX rats [142]. However, rats fed 3 g/kg Tualang honey showed the least lumbar calcium density with respect to the sham group [143]; it is possible to hypothesize that the high honey dosage led to diabetes induction in the rats, which, in turn, promoted bone loss.

Hasib et al. [144] found that honey administration (1 g/kg, 2 g/kg, and 4 g/kg for 2 weeks) showed a beneficial action on osteoporotic fracture healing by stimulating osteoblastogenesis, as demonstrated by the increased ALP level in sera.

Evaluating the fracture-healing properties of hydroxyapatite granules and honey, it was shown that the rats treated with honey alone displayed the lowest healing throughout the treatment period. Histological studies displayed that the group treated with hydroxyapatite alone had the weakest bone marrow formation with respect to all other treatment groups. Consequently, a hydroxyapatite and honey combination exerted better healing effects with respect to using them separately [145].

Hajizadeh et al. used a mandibular bone defect healing model in rats [146] to study the bone healing effects of honey. The defect was completed with sterile honey in the experimental group, whereas it remained unfilled in the control group. At the end of the experiment, histomorphometric studies showed that new bone formation was significantly better in the honey group than the control group after two and four weeks, thus showing that honey enhances the bone repair of small mandibular defects [146].

Ooi et al. explored in rats the joint effects of eight-week jumping honey supplementation and exercise on bone health [147]. Thirty min before the jumping exercise, 1 g/kg body weight/day honey was orally supplemented. The joint treatment developed major outcomes on the tibial mechanical properties and bone geometry with respect to the honey supplementation or jumping exercise alone.

Using the same dose of Tualang honey in combination with the jumping exercise in female rats for 8 weeks [148,149], or 16 weeks [150], Tavafzadeh et al. explored its role in bone health. In the first study, the authors reported that the joint combination of the jumping exercise and honey exerted more positive effects on the tibia and femur. In an additional study, the combined regime decreased bone resorption as sustained by the lower serum CTX levels [149]. Tavafzadeh et al. demonstrated that 16 continuous weeks of combined honey supplementation and the jumping exercise led to more effects on the tibia [150]. Interestingly, the positive effects of combinational therapy on the tibia could even be retained eight weeks after termination of the combined treatment [150].

Mosavat et al. explored the consequences of high and low-intensity jumping exercises combined with honey on bone [151]. They reported that the joined treatment causes a major improvement in the femoral and tibial bone mass, as well as ALP levels.

#### 3.1.2. In Vivo Human Studies

Few studies have been conducted on the human population, based on the evidence gained from cellular and animal models; see Table 3. In 2012, a randomized controlled trial on 79 healthy postmenopausal women failed to demonstrate any difference in bone density after 4 months of 20 g daily supplementation of honey versus hormonal replacement therapy, but the study was limited by the small sample size and short duration, as changes in bone mass usually occur at a slow rate [152]. Three different studies investigated the effects of combined aerobic dance exercise and honey supplementation [153,154,155]. The first was conducted on young women: the combined aerobic dance exercise and supplement of 20 g/day honey showed the greatest effect in increasing serum BALP levels compared to the honey supplement or aerobic exercise alone [153]. In another study on 46 young women, after 8 weeks of daily supplementation of 20 g honey and dance exercise, the highest increase in mean serum BALP and osteocalcin concentrations was observed, with a significant reduction in CTX serum levels [154]. In older women aged 25 to 40 years old, daily supplementation of 20 g honey for 8 weeks elevated serum total calcium level, whereas aerobic dance sessions alone increased cross-linked carboxyterminal telopeptide of type I collagen (1CTP). However, bone resorption was mitigated by honey supplementation, as shown in the reduction in 1CTP in the combined honey + exercise group (+14.75%) [155].

### 3.2. Tea

Tea is a product obtained from the plant *Camellia sinensis* and is one of the world’s most popular beverages; it has been hypothesised that it has bone-promoting properties due to its great content of flavonoids [156]; see Table 2.

#### 3.2.1. In Vitro and In Vivo Animal Studies

The osteoprotective effects of black tea, green tea, and their flavonoids have been demonstrated in OVX animal models; see Figure 1 and Figure 2. In detail, Das et al. [157,158,159] reported that black tea extract solubilized in water has preservative and restorative effects against OVX-induced bone loss in rats, as demonstrated by the reduced OC number and bone turnover rate, and by the enhanced bone mineral content and strength. Das et al. [160] showed that in OVX rats, black tea extract can be a prospective adjunct for calcium supplements to counteract early menopausal bone loss. Black tea extract’s effectiveness in preserving bone health is similar to that of 17 β-estradiol, possibly due to its phytoestrogenic efficacy [160]. Shen et al. reported that 14-month-old OVX and sham female rats treated with green tea polyphenols (GTP) in drinking water for 16 weeks displayed moderated bone loss and bone microarchitecture deterioration, leading to improved bone strength [161,162]. These beneficial effects are due to the enhanced antioxidant capacity and decreased oxidative stress DNA damage intrinsic to GTP [162,163,164].

Alternatively, Karmakar et al. [165] reported that black tea extract significantly improved the high-fat diet-induced skeletal alterations in rats.

In androgen-deficient aged rats, a male osteoporosis model, Shen et al. [166] demonstrated that GTP supplementation in drinking water decreased cortical and trabecular bone loss through the enhanced bone formation and suppressed bone resorption associated with GTP antioxidant properties.

In a systemic chronic inflammation LPS-induced bone loss model of adult rats, Shen et al. reported that GTP supplementation for 12 weeks caused higher femoral BMD, BMC, and serum osteocalcin, together with lower levels of serum TRAP, as a consequence of enhanced bone strength [167,168].

In a high-fat diet-induced bone deterioration model, Shen et al. [169,170] reported that GTP supplementation resulted in increased BMD, bone microarchitecture, and strength in obese rats by suppressing bone erosion and formation. Interestingly, in a binge alcohol-induced bone deterioration model, GTP supplementation in the drinking water increased femoral BMD and tibial cortical thickness at the mid-diaphysis through suppressing bone turnover rate [171].

Several groups have explored the osteoprotective effects of green tea on alveolar bone resorption periodontal disease models by suppressing inflammation and consequently osteoclastogenesis [172,173,174].

However, it is important to pay attention to the demonstration that at high doses, green tea extract may act as a pro-oxidant, damaging bone, as demonstrated by Iwaniec et al. [175]: in growing male mice, supplementation of green tea extract (1% and 2%, wt/wt in diet, mixed homogenously into the powdered diet) for 6 weeks was detrimental for bone growth with shorter bone length, decreased cortical bone volume and thickness, and altered BMC.

#### 3.2.2. In Vivo Human Studies

Many studies have been conducted, showing both positive and negative effects on bone health [176,177,178,179]; see Table 3. This is probably due to the high heterogeneity of the enrolled subjects, and differences in the doses and types of tea used and the duration of the supplementation.

Overall, a meta-analysis including 40 studies on the effect of tea consumption on bone health (15 cross-sectional studies, 14 cohort studies, and 12 case–control studies, which included 893,041 participants and 54,824 cases) revealed a positive correlation between tea consumption and BMD, and an inverse correlation between the risk of osteoporosis and fracture. Among tea-drinkers, BMD was significantly increased (SMD: 0.332, 95%CI 0.207–0.457), the risk of fractures of the hip, femur, and lumbar spine was reduced (RR = 0.910, 95%CI 0.845–0.980), and the risk of osteoporosis was reduced (RR = 0.800, 95%CI 0.674–0.950) [180].

### 3.3. Dried Plums

Several studies have recently linked increased consumption of fruits and vegetables with improved bone health, particularly with higher BMD and BMC, in a middle-aged population [181], in premenopausal [182] and postmenopausal women [183,184], and in the pediatric population [183,185,186,187,188].

Prunes (*Prunus domestica L*., also known as dried plums) have gained increasing attention among functional foods and plant-derived compounds with effects on bone health [189]. Prunes are a rich source of potassium, boron, copper, vitamin K, and phenolic compounds, such as chlorogenic acids, phenolic acids, and flavonoids [190,191], and dried plums are even richer in phenolic compounds than fresh ones [192]; see Table 2.

#### 3.3.1. In Vitro and In Vivo Animal Studies

Different in vitro studies have reported the potential of dried plums to prevent free radical damage and inflammatory responses in RAW 264.7 cells and MC3T3 [193,194,195]; see Figure 1 and Figure 2. Purified polyphenols from dried plums powder inhibit in vitro osteoclastogenesis through the downregulation of RANKL, NFATc1 in RAW264.7 following treatment with H_2_O_2_ or lipopolysaccharide [195]. Moreover, dried plum polyphenols (DPP) increase osteoblastogenesis in vitro in control conditions, and after TNF-α treatment [193]. Dried plums or their components improve bone quality, thus providing helpful strategies to counteract the damage to the structural integrity caused by radiotherapy or exposure to space radiation associated with long duration spaceflight [196]. Furthermore, dried plum (DP, 25% by weight) attenuates age-related bone loss as an anti-resorptive in different disease models. In detail, dried plum dietary supplementation prevented the damaging effects of estrogen deficiency on bone density and trabecular microarchitecture [197] and rescued bone loss in this same model [198]. These improvements in bone mass and architecture in the OVX female rat are due to the increased bone formation associated with insulin-like growth factor (IGF)-I and reduced bone resorption [199]. Moreover, dried plum dietary supplementation has strong effects on bone metabolism and prevents bone mass and microarchitecture deterioration in an orchidectomy model [200].

#### 3.3.2. In Vivo Human Studies

Different studies in postmenopausal women have shown positive effects of dried plums on bone health [201,202,203]. The first human study was conducted in 2002 on 58 postmenopausal women and showed that daily consumption of 100 g of prune for 3 months significantly increased the serum concentrations of BALP [201]. In another study on 100 menopausal women, daily consumption of 100 g of prunes for 1 year improved BMD of the ulna and lumbar spine in comparison to controls (*p* = 0.05), and both groups did not lose bone in comparison to the baseline [202]. Another study was performed on 160 postmenopausal women with mild bone loss, randomly assigned to the treatment group (dried plum 100 g/day) or control, and all provided with Calcium (Ca) and vitamin D for 12 months; in the dried plum group, a greater increase in the BMD of the ulna and spine was observed (*p* < 0.05). Furthermore, differences between the two groups were observed among serum levels of RANK, OPG, and sclerostin, although they were not statistically significant. These results showed a possible role for the OPG, RANKL, and sclerostin pathways in increasing BMD in dried plum consumers [203]

Prune consumption at 50 g/day and 100 g/day for 6 months in a group of 48 women with bone loss prevented loss of total BMD as indicated by no net change from baseline in total body BMD (*p* < 0.05), whereas the control group continued to lose bone; TRAP-5b, a marker of bone resorption, decreased at 3 months in both groups consuming dried plums (50 g *p* < 0.01, 100 g *p* < 0.04) [204].

In a randomized control trial on 235 postmenopausal women, the authors demonstrated that a 50 g daily dose of prunes could prevent loss of total hip BMD in postmenopausal women after 6 months, which persisted for 12 months. In addition, hip fracture risk (FRAX) worsened in the control group at 6 months compared with the baseline (+0.5 ± 0.5%, *p* < 0.05), but was maintained in the prune groups [205].

Recently, the role of dried plums on bone health was also studied in 35 men with some degree of bone loss aged 55 to 80: consumption of 100 g prunes for 3 months led to a significant decrease in serum osteocalcin (*p* < 0.001), while consumption of 50 g led to significant decreases in serum OPG (*p* = 0.003) and serum osteocalcin (*p* = 0.040), and an increase in the OPG:RANKL ratio (*p* = 0.041) [206]. All studies are reported in Table 3.

### 3.4. Blueberry

Blueberries are a rich source of polyphenols, and anthocyanins account for up to 60% of them; see Table 2. Thanks to this, blueberries are one of the richest sources of anthocyanins among common fruits, and it has been hypothesized that their health-promoting anti-inflammatory and antioxidant properties can be mainly attributable to these compounds. In vitro and in vivo studies (both on animal models and humans) support its beneficial effects in different cells, including bone health; see Figure 1 and Figure 2.

#### 3.4.1. In Vitro and In Vivo Animal Studies

The first study was performed using an ovariectomized rat model. Thirty 6-month-old female Sprague-Dawley rats were either sham-operated (Sham) or OVX and divided into three groups: Sham, OVX (control), Ovx+blueberry (5% blueberry *w*/*w*) with a 100-day duration of treatment. The authors demonstrated that OVX rats developed approximately 6% loss of whole-body, tibial, femoral, and 4th lumbar BMD. Blueberry treatment was able to prevent the loss of whole-body BMD and had an intermediary effect on the prevention of tibial and femoral BMD when compared to either Sham or OVX controls. Blueberry’s bone-protective action can be linked to the inhibition of OVX-induced increase in bone turnover, as shown by decreased femoral mRNA levels of TRAP, collagen type I, and BALP to the Sham levels. All these results highlight that blueberry can protect against bone loss [207]. Chen et al. conducted in vitro studies using Phenolic acids (PAs) as metabolites derived from polyphenols that can be measured in the sera of rats fed a blueberry-rich diet. PAs stimulated OB proliferation and differentiation, and inhibited adipogenesis. To deepen the mechanisms, the same authors injected hippuric acid, one of the major metabolites detected in animal sera following blueberry consumption, to prepubertal female mice for 2 weeks. This compound resulted in a bone mass increase by promoting OB activity. These PA effects arose from the activation of G-protein-coupled receptor 109A, p38 MAPK, as well as osterix [208].

Chen et al. also studied the effects of a particular blueberry-associated serum PA, 3-(3-hydroxyphenyl)-propionic acid (PPA), on senescence signaling and osteoblastogenesis. Four doses of PPA (0.1, 0.5, 1, and 5 mg/kg/day; daily i.p.) were injected into 1-month-old female C57BL6/J mice for 30 days. The authors demonstrated significantly higher bone volume, trabecular thickness, and enhanced OB number, together with a reduced OC number in PPA-treated groups with respect to the controls. These outcomes were associated with changes in bone formation markers in sera and bone marrow plasma. In bone PPA, injection decreased senescence signature levels, such as senescence-associated β-galactosidase activity, PPARγ, p21, and p53 [209].

Zhang et al. examined the effects of three different levels of blueberry diet supplementation (1, 3, and 5%) for 35 days on bone quality using female rats. They reported that BMD and BMC were dose-dependently augmented in blueberry-fed rats with respect to the controls. The levels of the pro-osteoclastogenic cytokine RANKL dose-dependently reduced in the femur of blueberry animals. Furthermore, PPARγ expression was inhibited in blueberry diet rats with respect to controls. The same authors also showed in vitro that the blueberry diet rat serum inhibited RANKL expression, primarily in mesenchymal stromal cells, but also in mature OBs, osteocytes, and pre-adipocytes. These results imply that bone resorption inhibition may have a role in the augmented bone mass occurring during early development following blueberry consumption [210].

Domazetovic et al. explored the role of Blueberry juice (BJ), from Vaccinium myrtillus, rich in polyphenols, as an antioxidant and anti-osteoclastogenic in MLO-Y4 osteocytes. In detail, BJ avoids oxidative stress-induced apoptosis and reverses the RANKL and sclerostin increase in MLO-Y4. BJ also prevents oxidative stress-induced cytotoxicity in bone marrow mesenchymal stromal cells. The authors reported that the blueberry dry extract with equal amounts of total soluble polyphenols exerted the same effects compared with BJ. They also reported that blueberry works as both an antioxidant and an activator of sirtuin type 1, a class III histone deacetylase regulating cell death, blocking bone resorption [211].

In another study, (2020) Domazetovic et al. found in human glutathione-depleted SaOS-2 cells that BJ, containing 7.5 or 15 μg∙mL^−1^ total soluble polyphenols, can thwart oxidative stress-induced inhibition of osteoblastogenesis. Their findings suggest that BJ provides protection against factors associated with oxidative damage during bone remodeling and formation, upregulating BALP and RUNX2 levels. These effects are mediated by the activation of sirtuin type 1 deacetylase [212].

Cladis et al., in 2022, studied the effect of blueberry polyphenols on bone fragility. Five-month-old ovariectomized Sprague-Dawley rats (n = 10/gp) were fed a purified extract of blueberry polyphenols (0–1000 mg total polyphenols/kg bw/day) or lyophilized blueberries (50 mg total polyphenols/kg bw/day) for 90 days. They found that blueberry polyphenols weakly influence BMD and bone mechanical properties. The authors reported different biases, including the small number of animals used [213].

#### 3.4.2. In Vivo Human Studies

Despite the large amount of pre-clinical evidence, only one clinical trial has been conducted with the aim of studying the effect of blueberries on 13 healthy postmenopausal women. Participants consuming low (17.5 g/d) and medium (35 g/d) doses of blueberries for 6 weeks retained significantly more calcium in bone compared with no treatment (calcium retention +5.6%, *p* < 0.01 and 4.5%, *p* < 0.05, respectively). The medium dose of blueberry powder reduced serum concentrations of RANKL by 14% (*p* < 0.05), and all doses of blueberry powder decreased concentrations of serum P1NP by a mean of 26% (*p* < 0.01). No statistically significant relationships were found between the treatment and RANKL/OPG ratio, and markers of bone resorption (sclerostin, OPG, CTX-II, urinary NTx normalized to creatinine) [214]; see Table 3.

### 3.5. Olive Oil

Extra virgin olive oil (EVOO) is one of the main components of the Mediterranean Diet, a food regimen with well-known properties for health in general, and specifically in bone status maintenance [215]. Many of the beneficial effects of the Mediterranean Diet (MD) can be ascribed to EVOO [216], thanks to its special composition of unsaturated fatty acid and phenolic compounds that act synergistically [217]; see Table 2.

#### 3.5.1. In Vitro and In Vivo Animal Models

In 6-month-old OVX rats with induced inflammation, diet supplementation with 50 g/kg EVOO for 80 days avoided a reduction in femoral, metaphyseal and diaphyseal BMD, compared with those without inflammation. Furthermore, in ovariectomized rats with inflammation, EVOO augmented the failure load of femurs. However, the levels of plasma osteocalcin and urinary deoxypyridinoline were not significantly modified by the treatment. Other authors supplemented 12–14-month-old ovariectomized rats with EVOO (1 mL/kg body weight) for 12 weeks (4 weeks before and 8 weeks after ovariectomy). The treatment avoided a decrease in the trabecular and cortical bone thickness. Furthermore, it prevented calcium mobilization from bone, as shown by a decreased plasma calcium concentration in the treated group with respect to the OVX control [218].

The efficacy of OO (Olive Oil) supplementation (1 mL/100 g diet) and diethylstilbestrol (25 ug/kg diet), a synthetic estrogen, for 12 weeks was evaluated in 6-month-old OVX rats. The BMD of the left femur and lumbar spine of the OVX rats was enhanced by both treatments, possibly due to the oxidative stress reduction in the supplemented groups. Thus, this study demonstrated that OO was as useful as estrogen replacement therapy in the prevention of postmenopausal bone loss [219].

In addition, diet supplementation with the phenolic-rich extract of EVOO (800 mg/kg) for 12 weeks exerted estrogenic effects in 12-month-old OVX rats. However, this treatment did not prevent bone loss induced by OVX [220], which can be related to a shorter treatment period.

Olive plants have different polyphenols, such as tyrosol, hydroxytyrosol, and oleuropein [221,222]. Each of these molecules or their mixture can defend against bone loss. It has been reported that in MC3T3-E1, hydroxytyrosol, tyrosol, and oleuropein did not affect the proliferation or the levels of collagen and ALP, but interestingly, oleuropein significantly augmented calcium deposition in vitro. Hydroxytyrosol also showed a similar outcome without reaching statistical significance [223]. In addition, oleuropein significantly reduced osteoclastogenesis from a spleen culture. At high doses, oleuropein strongly suppressed OC formation. Hydroxytyrosol and tyrosol also decreased osteoclastogenesis in vitro, and oral administration prevented trabecular bone loss in OVX rats [223]. The phenolic extract of different olive oils can enhance the proliferation of the human MG-63 osteosarcoma cell line. However, this effect was independent of the OO phenolic content [224]. Differently, MG-63 cells treated with distinct hydroxytyrosol, p-coumaric acid, caffeic acid, luteolin, ferulic acid, and apigenin enhanced OB proliferation. However, the total phenolic content of VOO (Virgin olive oil) from numerous olive species showed greater effects than individual phenolic acid, thus suggesting synergistic effects among phenolic contents on OB proliferation. However, this effect was better using extracts from unripe fruits than ripe fruits due to the elevated phenolic index of the former [225]. Santiago-Mora et al. reported that oleuropein (1 µM and 100 µM) increased osteoblastogenesis in human bone marrow culture by the upregulation of RUNX2, osterix, collagen I, BALP, and osteocalcin with a consequent increase in mineral deposition and inhibited adipogenesis. This was also associated with an enhancement in the OPG/RANKL ratio following oleuropein treatment. Conversely, expression of pro-adipogenic genes, such as peroxisome proliferator-activated receptor gamma 2 (PPARγ2), fatty acid-binding protein 4 (FABP4), and the lipoprotein lipase (LPL), was inhibited by oleuropein. Adipogenesis was significantly decreased in oleuropein-treated cultures [226].

In 6-month-old inflamed OVX rats, oleuropein treatment (added to the diet) for 100 days prevented a reduction in femoral BMD. However, oleuropein treatment did not increase bone biomechanical strength [227]. Conversely, it has been reported that bone strength was only changed by a major dose of oleuropein [228]. Hagiwara et al. treated OVX mice with hydroxytyrosol, oleuropein or tyrosol at 10 mg/kg orally for 28 days. Theoleuropein and hydroxytyrosol managements enhanced the femoral trabecular BMD. Hydroxytyrosol efficacy was superior with respect to oleuropein and this effect was associated with a major hydroxytyrosol absorption in vivo [223]. Olive mill wastewater arises from olive oil production and is rich in polyphenols. Puel et al. fed 6-month-old inflamed ovariectomized rats for 84 days with a diet supplemented with tyrosol, or hydroxytyrosol, or olive mill wastewater, or two different concentrations of mill wastewater extract. Supplementation of hydroxytyrosol, tyrosol, and two concentrations of olive mill wastewater extract avoided a decrease in femoral BMD. However, bone biomechanical strength was not modified by any of the treatments [221].

#### 3.5.2. In Vivo Human Studies

Many studies have investigated the association between olive oil consumption in the diet and bone health; see Table 3. In a cohort of 523 women, people who declared in an FFQ a higher dietary intake of olive oil (>18.32 g/day) had significantly higher volumetric bone mineral density (vBMD, *p* < 0.01), total, trabecular, and cortical bone density compared with those with a lower intake of olive oil [229].

Randomized controlled trials have been performed on different populations, showing a positive effect of olive oil on bone markers. One study on one hundred and twenty-seven men aged 55–80 years showed that after 2 years of follow-up, people following MD enriched with VOO had an increase in total osteocalcin (*p* = 0.007) and P1NP (*p* = 0.01), while CTX decreased significantly in all study groups (*p* = 0.0001) [230].

The PREDIMED trial, a large, multicenter, randomized and controlled parallel group trial conducted on 870 participants (males aged 55–80 years and females aged 60–80 years), failed to demonstrate a reduced fracture risk in people assigned to an MD supplemented with 50 g or more per day of EVOO in comparison with controls, whereas a non-significant trend to a lower risk was also observed for total olive oil consumption; the median of intervention was 5.2 years [231]. However, individuals with higher EVOO consumption (mean consumption of total olive oil 56.5 g/day) showed a 51% reduction in the risk of osteoporosis-related fractures compared to those with lower consumption, proving that higher consumption of EVOO in the diet reduces the risk of osteoporosis-related fractures [231].

**Table 2:** Main components and effective dosage of functional foods on human bone health.

Functional Food	Components	Effective Dosage	References
Honey	Flavonoids, phenolic acids, vitamin D3	20 g/day + aerobic exercise	[153,154,155]
Tea	Flavonoids	Not available	[178]
Dried plums	Chlorogenic acids, phenolic acids, and flavonoids	100 g/day	[156,201,202,203,204,205,206]
Blueberry	Anthocyanins	35 g/day	[200]
Extra virgin olive oil	Unsaturated fatty acid and phenolic compounds	50 mL/day + Mediterranean Diet	[230]

**Table 3:** Interventional trials on nutraceutical and functional food consumption and bone health in humans.

Nutraceutical/Functional Food	Ref.	Study Design/Methods	Study Population	Test Report							Intervention Duration	Effect of Nutraceuticals/Functional Foods on Bone Health
				Active Groups						Control Groups		
Resveratrol	[39]	Randomized double-blind placebo-controlled trial RSV_low_ (n = 23) RSV_high_ (n = 25) Placebo (n = 26)	74 M obese men with metabolic syndrome, mean age 49.3 ± 6.3 y, mean BMI 33.7 ± 3.6 kg/m	RSV_high_: 1.000 mg/day RSV		RSV_low_: 150 mg/day RSV				Placebo	16 weeks	↑ BALP and LS vBMD_trab_ dose-dependently with RSV, positive correlation in BALP and LS vBMD_trab_
Resveratrol, Isoflavones aglycones, and Equol	[40]	Randomized, placebo-controlled trial Active (n = 38) Control (n = 38)	76 F healthy postmenopausal, 50–55 y, ≥ 12 months of cessation of menses	Dietary supplement containing 200 mg of fermented soy (including 80 mg of isoflavone aglycones and 10 mg of equol) and 25 mg of resveratrol from Vitis vinifera						Placebo	12 months	↓ DPD TRACP-5b, ↑ osteocalcin, BALP compared to placebo
Resveratrol	[41]	Randomized, double-blind, placebo-controlled, two-period crossover intervention Active (n = 63) Control (n = 66)	125 F healthy postmenopausal, age 45–85 y, ≥12 months of cessation of menses	75 mg twice/day (total 150 mg) RSV						Placebo	12 months	↑ BMD lumbar spine and neck of femur, ↓ CTX, compared with placebo
Curcumin	[67]	Randomized, double-blind trial. Alendronate (n = 20) Alendronate + curcumin (n = 20) Control (n = 20)	60 F postmenopausal with osteoporosis, age 55–65 y, ≥5 y cessation of menses	Alendronate 5 mg/day + Calcium carbonate 1.000–1.500 mg/day		Alendronate 5 mg/day + Curcumin 110 mg/day + Calcium carbonate 1.000–1.500 mg/day				Calcium carbonate 1.000–1.500 mg/day	12 months	Curcumin + alendronate group ↓ BALP and CTX; ↑ BMD in four areas compared to the control and alendronate groups
Curcumin	[68]	Randomized, blind, placebo-controlled trial. Active (n = 50) Control (n = 50)	100 adult patients with spinal cord injury trauma within the previous 6 months and paraplegia or quadriplegia, age 19–65 y	110/mg/kg/day Curcumin						Placebo	6 months	In curcumin group ↑ BMD compared with the beginning and compared to controls
β-carotene	[88]	Intervention study to test the efficacy of high-dose retinol and BC supplements for reducing the risk of mesothelioma and lung cancer in a risk group	2322 adults previously exposed to crocidolite (blue asbestos)	30 mg/day BC for 6 y; after that, 7.5 mg RE/day		0.75 mg/day BC for 6 y; after that, 7.5 mg RE/day				7.5 mg/day RE as retinyl palmitate	1–16 years	In M, cumulative dose of BC was associated with ↓ risk of any fracture and osteoporotic fracture
β-cryptoxanthin	[109]	Interventional trial	21 adults (10 M, 11 F), 23–47 y	192 mL/day of reinforced juice prepared from Satuma mandarin containing 2880 μg of β-cryptoxanthin						192 mL/day of juice prepared from Satuma mandarin containing 1540 μg of β-cryptoxanthin	56 days	Juice consumption ↑ γ-carboxylated OC; reinforced juice ↓ TRAP and NTx
β-cryptoxanthin	[110]	Interventional trial	90 healthy adults, 19 M and 71 F (35 premenopausal and 36 postmenopausal)	Satsuma mandarin juice at concentration of 1.5 mg/200 mL	Satsuma mandarin juice at concentration of 3 mg/200 mL				Satsuma mandarin juice at concentration of 6 mg/200 mL			↑ ALP and γ-carboxylated osteocalcin and ↓ TRAP and NTx
Honey	[152]	Randomized controlled trial Honey group (n = 40) HRT (n = 39)	79 healthy F postmenopausal, ≥12 months of cessation of menses, age 45–60 y	20 g/day of Tualang honey						HRT (Femoston^®^) contain 1 mg Estradiol valerate and 5 mg Dydrogesterone	4 months	No differences
Honey	[153]	Interventional trial Ex (n = 10) H (n = 10) HEx (n = 10) C (n = 10)	40 healthy F, 19–28 y	Ex: aerobic dance exercise * (1 h per session, 3 sessions per week)	H: 20 g/day Malaysian local Gelam honey diluted in 300 mL of plain water			HEx: aerobic dance exercise * +20 g/day Malaysian local Gelam honey diluted in 300 mL of plain water (30 min before exercise)		C: Sedentary and no honey supplementation	6 weeks	In H and HEx groups: ↑ALP HEx group exhibited the highest percentage ↑ALP.
Honey	[154]	Randomized controlled trial 8Ex8S (n = 12) 8H8S (n = 12) 8ExH8S (n = 12) 16S (n = 12)	48 healthy F, 19–25 y	8Ex8S: aerobic dance exercise * (1 h per session, 3 sessions per week)	8H8S: 20 g Tualang honey diluted in 300 mL of plain water			8ExH8S: aerobic dance exercise * +20 g/day Tualang honey diluted in 300 mL of plain water (30 min before exercise)		16S: 16 weeks of sedentary and no honey supplementation	8 weeks of intervention followed by 8 weeks of sedentary lifestyle	After intervention (8 w) in 8E×H8S: ↑ ALP, osteocalcin and ↓1CTP. After 8 weeks of cessation of intervention in 8E×H8S: ↑ serum total calcium, ALP, TAS, GSH
Honey	[155]	Interventional trial Ex (n = 11) H (n = 11) HEx (n = 11) C (n = 11)	44 healthy F, 25–40 y	Ex: aerobic dance exercise * (1 h per session, 3 sessions per week)	H: 20 g/day Malaysian local Gelam honey diluted in 300 mL of plain water			HEx: aerobic dance exercise * +20 g/day Malaysian local Gelam honey diluted in 300 mL of plain water (30 min before exercise)		C: sedentary and no honey supplementation	8 weeks	H: ↑ serum total calcium level Ex: ↑ 1CTP HEx: ↓ 1CTP (bone resorption is mitigated by honey supplementation)
Tea	[178]	Randomized, double-blind, placebo-controlled clinical trial Active (n = 61) Control (n = 60)	121 F postmenopausal, 50–70 y, BMI ≥ 25 kg/m^2^	Decaffeinated green tea extract containing 843 mg (2)-epigallocatechin-3-gallate in 4 capsules/day						Placebo in 4 capsules/day	12 months	No effects
Dried plums	[201]	Randomized controlled clinical trial	58 F postmenopausal	100 g/day dried plums						75 g dried apples/day	3 months	↑ IGF-I and BALP
Dried plums	[202]	Comparative control randomized study Dried plums (n = 55) Dried apples (n = 45)	110 F, 1–10 years postmenopausal with mild bone loss	100 g/day dried plums + 500 mg/day Ca + 400 IU/day vitamin D						75 g/day dried apples + 500 mg/day Ca + 400 IU/day vitamin D	12 months	↑ BMD of ulna and spine in comparison with dried apple; ↓ BALP, TRACP 5b in comparison with corresponding baseline values
Dried plums	[203]	Randomized controlled trial Dried plums (n = 55) Dried apples (n = 45)	110 F, 1–10 years postmenopausal with mild bone loss	100 g/day dried plums + 500 mg/day Ca + 400 IU/day vitamin D						75 g/day dried apples + 500 mg/day Ca + 400 IU/day vitamin D	12 months	Dried plums: in comparison with corresponding baseline values, not statistically relevant ↑ RANKL, OPG, ↓ sclerostin
Dried plums	[204]	Randomized controlled trial 100 gDP (n = 16) 50 gDP (n = 16) Control (n = 16)	48 F postmenopausal with mild bone loss, 65–79 y	100 g/day dried plums + 500 mg/day Ca + 400 IU/day vitamin D			50 g/day dried plums + 500 mg/day Ca + 400 IU/day vitamin D			75 g/day dried apples + 500 mg/day Ca + 400 IU/day vitamin D	6 months	Both doses of dried plum: no changes in BMD (whereas control group ↓ BMD). ↓ TRAP-5b at 3 months
Dried plums	[205]	Randomized controlled trial 100gDP (n = 78) 50gDP (n = 79) Control (n = 78)	235 F postmenopausal, 62.1 ± 5.0 y with a BMD T-score of <0.0 and >−3.0 at any site	100 g/day prunes + supplemented as necessary to meet the daily intake of 1200 mg calcium carbonate and 800 IU vitamin D3 (diet + supplements)			50 g/day prunes + supplemented as necessary to meet the daily intake of 1200 mg calcium carbonate and 800 IU vitamin D3 (diet + supplements)			Supplemented as necessary to meet the daily intake of 1200 mg calcium carbonate and 800 IU vitamin D3 (diet + supplements)	12 months	50 g prune group: no changes in BMD (whereas control group ↓ BMD); in both prunes dosages no changes in FRAX (worsened in controls)
Dried plums	[206]	Randomized trial Group A (n = 15) Group B (n = 12) Group C (n = 8)	35 M, 55–80 y with a BMD t-score 0.1–2.5 SD	Group A: 100 g/day prunes + multivitamin containing 800 IU/day vitamin D and 450 mg/day calcium			Group B: 50 g/day prunes + multivitamin containing 800 IU/day vitamin D and 450 mg/day calcium			Multivitamin containing 800 IU/day vitamin D and 450 mg/day calcium	3 months	100 g prunes: ↓ osteocalcin; 50 g: ↓ OPG and osteocalcin, ↑ OPG:RANKL ratio
Blueberries	[124]	Double-blind randomized crossover trial	13 F, 45–70 y, >4 y post natural menopause or total hysterectomy	Randomly assigned to a sequence of 3 intervention periods, each corresponding to a low (17.5 g/d), medium (35 g/d), or high (70 g/d) dose of freeze-dried BB powder							6 weeks	17.5 g/d and 35 g/d: ↑ calcium retention; 35 g/d ↓ RANKL; all doses ↓ P1NP
Olive oil	[230]	Randomized trial MedDiet + VOO (n = 42) MedDiet+nuts (n = 51) Controls (n = 34)	127 M 55–80 y with diagnosis of type 2 diabetes or at least three cardiovascular risk factors (hypertension/dyslipidemia/BMI ≥ 25 kg/m) or a family history of premature cardiovascular disease	MedDiet + VOO: Mediterrean Diet + at least 50 mL/day of extra virgin olive oil			MedDiet + nuts: Mediterrean Diet + 30 g/day mixed nuts (walnuts, almonds, and hazelnuts)			Advice on low-fat diet	2 years	MedDiet+VOO: ↑ osteocalcin and P1NP ↓ CTX in all study groups
Olive oil	[231]	Multicenter, randomized controlled parallel group trial	870 participants, M 55–80 y and F 60–80 y with diagnosis of type 2 diabetes or at least three cardiovascular risk factors (hypertension/dyslipidemia /BMI ≥ 25 kg/m)	MedDiet + VOO: Mediterrean Diet + at least 50 mL/day of extra virgin olive oil			MedDiet + nuts: Mediterrean Diet + 30 g/day mixed nuts (walnuts, almonds, and hazelnuts)			Advice on low-fat diet	Median of 5.2 years of intervention	−51% in the risk of osteoporosis-related fractures in individuals with the highest EVOO consumption (mean 56.5 g/day)

Abbreviations: ↑: increase, ↓: decrease; * 1 h per session, 3 sessions per week; BALP, Bone Alkaline Phosphatase; BB, Blueberry; BC, β-carotene; BMI, Body Mass Index; CTX, C-terminal telopeptide type-1 collagen levels; DP, dried plums; DPD, deoxypyridinoline; F, female; FRAX: Hip fracture risk; HRT, Hormonal replacement therapy; LS vBMD_trab_, lumbar spine trabecular volumetric bone mineral density; M, male; NTx, N-telopeptide of type I collagen; OPG, osteoprotegerin; P1NP, Procollagen type 1 N propeptide; RE, retinol equivalents; RSV, resveratrol; TAS, Total antioxidant status; TRAP, Tartrate-resistant acid phosphatase; TRACP 5b, Tartrate-resistant acid phosphatase 5b; VOO: Virgin olive oil.

## 4. Methods

To write this review, we used Pubmed as the database, with the following search words: Functional foods, honey and bone, tea and bone, dried plums and bone, blueberry and bone, olive oil and bone. The publication time included papers published between 1997 and 2024.

## 5. Conclusions and Future Perspectives

This review reports the scientific and clinical evidence for the positive role of nutraceuticals and functional food in bone health, focusing both on in vitro molecular mechanisms, and in vivo animal studies and trials, in order to provide the beneficial effects of some nutraceuticals and functional foods with adequate clinical and experimental evidence useful to improve bone health in real life (Figure 1 and Figure 2, Table 3).

All the described nutraceuticals and functional foods modulated bone cell activity by decreasing osteoclast differentiation and increasing osteoblastogenesis, mainly affecting the oxidative stress and apoptotic signals. The effect on osteoclastogenesis can be direct, as a consequence of the interaction between the nutraceuticals and cells, but also indirect, as mediated by RANKL-reduced expression of osteoblasts. However, other mechanisms are also involved, as previously described, and all are responsible for the improvement in bone microarchitecture in the different animal models. The beneficial effects are also evident in human studies. All the in vivo models described also considered the effect of digestion and colonic metabolism. In detail, nutraceuticals and functional foods include different phytochemicals with high chemical diversity. Their activity in vivo is hard to demonstrate due to their mild physiological effects as well as the great inter-individual variability detected. Different polyphenols display a low bioavailability and arrive in the colon almost unaltered. Here they find the gut microbes, leading to a two-way interaction in which polyphenols affect the composition of gut microbiota, which in turn catabolizes the ingested polyphenols to produce different metabolites, which are often better absorbed and more active than the original polyphenols. In humans, the quantity and type of polyphenol metabolites generated depend on the function and composition of the gut microbiota. Furthermore, not all the metabolites displayed the same biological activity, and consequently, the final health effects of dietary polyphenols are linked to the composition of the gut microbiota [232].

All these findings lead us to conclude that the right nutrition with the inclusion of polyphenols, carotenoids, curcumin, PUFAs, as well as high quality functional foods provides a protective effect on bone health and surely on other tissues.

## Data Availability

Not applicable.
